# Extracellular vesicles from cancer cell lines of different origins drive the phenotype of normal oral fibroblasts in a CAF-like direction

**DOI:** 10.3389/fonc.2024.1456346

**Published:** 2024-09-24

**Authors:** Tine M. Søland, Aleksandra Lipka, Ann-Kristin Ruus, Ann-Kristin Molværsmyr, Hilde K. Galtung, Trude M. Haug

**Affiliations:** Institute of Oral Biology, Faculty of Dentistry, University of Oslo, Oslo, Norway

**Keywords:** oral cancer, oral squamous cell carcinoma, cancer-associated fibroblasts, phenotype, proliferation, migration, EV, OSCC

## Abstract

**Introduction:**

Normal oral fibroblasts (NOFs) are located in the connective tissue of the oral mucosa. The NOFs play an important role in wound healing, tumor progression, and metastasis. They are subjected to influence by external and internal stimuli, among them extracellular vesicles (EVs), that are considered as important players in cell to cell communication, especially in carcinogenesis and metastatic processes. During tumorigenesis, stromal NOFs may undergo activation into cancer-associated fibroblasts (CAFs) that modify their phenotype to provide pro-oncogenic signals that in turn facilitate tumor initiation, progression, and metastasis. The aim of the study was to reveal the effect of EVs derived from local (oral squamous cell carcinoma – OSCC) and distant (pancreatic adenocarcinoma – PDAC; malignant melanoma brain metastasis – MBM) cancer origin on NOFs and their possible change into a CAF-like direction.

**Methods:**

The effect of each of the cancer EV types on NOFs proliferation, viability, and migration was tested. Also, changes in gene expression of the well-established CAF biomarkers ACTA2, FAP, PDGFR, and two putative CAF biomarkers, the Ca2+- activated ion channels ANO1 and KCNMA, were studied.

**Results:**

Obtained results indicate that NOFs receive and process signals transmitted by EVs originating from both OSCC, PDAC, and MBM. The fibroblast response was dependent on EV origin and concentration, and duration of EV exposure.

**Conclusion:**

The present results indicate that the molecular cargo of the EVs direct NOFs towards a pro-tumorigenic phenotype.

## 1 Introduction

Oral squamous cell carcinoma (OSCC) is a devastating disease with a high mortality, where the 5-year disease-specific survival is about 60%. OSCC progression depends on a vivid crosstalk between the carcinoma cells and the non-neoplastic tumor stroma (1, 2). Here, a group of activated fibroblasts known as cancer associated fibroblasts (CAFs) is present and is generally assumed to be tumor-promoting (3, 4). Numerous studies have shown that CAF-rich OSCC is associated with significantly shorter patient survival. In particular, the abundant presence of CAFs in OSCC has been significantly correlated to poor overall survival and shortened disease-free survival in a recent meta-analysis (5). A stroma rich in CAFs is also positively associated with histological features such as depth of invasion, lymphatic invasion, and extra-nodal metastatic spread, all related to increased tumor aggressiveness and poor prognosis (6, 7). CAFs can have diversified origin, although most CAFs are thought to initiate from local fibroblasts experiencing tissue dysfunction (8, 9). CAFs heterogenous subpopulations include antigen-presenting CAFs (apCAFs), myofibroblastic CAFs (myCAFs), and inflammatory CAFs (iCAFs) (10). In sites of colorectal, liver, lung, and bone metastases, CAFs have been found to originate from resident fibroblasts, also known as metastasis-associated fibroblasts (MAFs). This may suggest an influence of metastatic cancer cells on resident fibroblasts, and a role of resident fibroblasts as contributors to the establishment of metastatic lesions and development of therapeutic resistance in metastatic tumors (11). Therefore, it is also of interest to investigate how EVs from cancer cells other than OSCC can influence oral fibroblasts.

Extracellular vesicles (EVs) are particles delimited by a lipid bilayer and secreted by most cell types, including cancer cells, into their extracellular surroundings (1, 12, 13). EVs exert their biological functions by delivering diverse signaling molecules such as proteins, miRNAs, other nucleic acids, and lipids to recipient cells. The EVs can modify recipient cells either by attaching to receptors bound to their plasma membrane, by fusing with their membrane followed by release of the EV content, or by being engulfed by the recipient cell. Due to the diverse EV content, the cellular response to EVs is also varied, and has been shown to affect the phenotype and activity of the recipient cells. This potential to modify the phenotype is extremely important in the case of cancer derived EVs that may change the environment to become more receptive for tumor growth and metastasis (1, 14–16).

An overall change of normal cell phenotype into a pathophysiological state can also be induced by dysregulation of Ca^2+^ signaling (17). This can be caused by changes in the activity of Ca^2+^ channels, Ca^2+^ pumps, Ca^2+^ binding proteins, as well as K^+^ and Cl^-^ channels, all of which are previously found to be differentially expressed in cancer cells. Additionally, a change of ion channel profile in the cells of the tumor microenvironment may further contribute to cancer progression (18). Not much is known about the role of ion channels in fibroblast activation and transformation into CAFs, but particularly Ca^2+^ channels have been in focus regarding development and function of cancer cells, due to the central role of Ca^2+^ signaling in cell cycle control, proliferation, and migration (19). Since similar changes in phenotype is experienced also during CAF development, one may expect similar changes in the ion channel profile as well. To the best of our knowledge, the influence of OSCC EVs on the ion channel or Ca^2+^ signaling profile in stromal cells has not previously been evaluated.

Although the general concept of tumor-stroma interaction is well established, the specific effects of EVs from OSCC cells on normal oral fibroblasts in tumor stroma are poorly understood (20, 21). Thereby, such studies are needed to better understand the role of OSCC-derived EVs in this interplay. Furthermore, it is also of interest to investigate the influence of EVs from cancer cells other than OSCC on normal oral fibroblasts to increase our knowledge of whether oral fibroblasts can receive and process signals from cancers of different origin and whether the fibroblast response is cancer type dependent. This is important for development of new therapeutic strategies for OSCC as well as for metastases to the oral cavity from distant tumors. Therefore, the aim of the present study was to investigate phenotype change and the expression of CAF markers in normal oral fibroblasts after exposure to EVs from cancer cell lines of different origin. As SCC is the most common malignancy in the oral cavity, we selected an OSCCcell line (OSCC), and also included a pancreatic adenocarcinoma cell line (PDAC) and a malignant melanoma brain metastasis cell line (MBM), both of human origin, as representatives of adenocarcinomas and melanomas with poor prognosis and frequent tumor metastases. We focused on relevant phenotypic traits such as migration and proliferation, as well as three of the most established CAF biomarkers smooth muscle actin (α-SMA) (also known as actin alpha 2 or ACTA2), fibroblast activation protein (FAP), and platelet-derived growth factor receptor (PDGFR). In addition, we investigated whether two Ca^2+^-activated ion channels (Ano1 and KCNMA), involved in cancer cell proliferation and invasion (17) were affected.

## 2 Materials and methods

### 2.1 Cell culture and EVs

Oral gingival fibroblasts (, a kind gift from Prof. D. Costea, University of Bergen, Norway; REK approval 2010/481) were grown in DMEM, high glucose, GlutaMAX™ Supplement (Gibco, Life Technologies, Paisley, UK) medium supplemented with 10% FBS (Merck, Sigma-Aldrich) and 1X Antibiotic Antimycotic Solution (penicillin, streptomycin, and amphotericin B (PSA), Merck Sigma-Aldrich, USA). Cells were grown at 37°C in a 5% CO_2_ atmosphere incubator. Oral gingival fibroblasts were isolated from clinically normal looking gingival areas of an individual patient. Based on the expression of CD146, the presence of mesenchymal stem cells in fibroblasts isolated from the gingiva does not exceed 5% (22).

The EV isolation and verification protocol has been described in detail elsewhere (23). Briefly, EVs were isolated from commercially available cell lines of human oral squamous cell carcinoma (OSCC; PE/CA-PJ49/E10; ECACC, Salisbury, UK), human pancreatic adenocarcinoma (PDAC; BxPC3; ATCC, Manassas, USA), and human melanoma brain metastasis (MBM; H3 (24), a kind gift from Prof. F. Thorsen, University of Bergen, Norway). Cells that had previously been gradually adapted to 1% exosome-depleted FBS in advanced culture media were grown in an Integra CELLine AD1000 culture flask. Following serial collection of the supernatant, a combination of ultrafiltration and size-exclusion chromatography was used for the isolation of EVs. The EVs were then characterized by nanoparticle tracking analysis, immunoaffinity capture, flow cytometry, Western blot, and transmission electron microscopy.

### 2.2 Confocal imaging of EV uptake by oral fibroblasts

The oral fibroblasts (passage 4-8) were cultured until confluence before they were trypsinated and centrifuged (5 min; 1000×g). Cells were resuspended in a starvation medium (0.5% exosome-depleted FBS) and a volume of 100 µL solution with ~ 5000 fibroblasts was seeded onto 24-well plate (within each well a coverslip was placed before seeding) and grown overnight in the cell incubator. To document EV uptake by fibroblast, 100 µl of EV stock solution from each cell line (in PBS; concentration of 10^11^ particles/mL) was stained separately with PKH67 Fluorescent cell linker kit (Merck Sigma-Aldrich, USA) according to the manufacturer’s protocol. Then, these EVs were added to the fibroblasts at a concentration of 10^8^ particles/mL. After 4 h, the cells were fixed with PFA (Paraformaldehyde, Sigma Aldrich, USA) and the coverslips were placed on the slides and mounted with Dapi-Fluoromount-G™ Clear Mounting Media (SouthernBiotech, USA) to visualize nuclei. Slides were analyzed using a confocal microscope (Leica TCS SP8) at the Advanced Light Microscopy Core Facility at the Oslo University Hospital (OUS).

### 2.3 Viability and proliferation assay

The fibroblasts were seeded at a density of 300 000 cells/well in a 6-well plate and incubated in 2 mL of the standard fibroblast growth medium for 24 h. The medium was then replaced with the starvation medium. Next, after 17 h, the starvation medium was replaced with 2 ml of the fresh starvation medium supplemented with different concentrations of each of the EVs (10^6^, 10^7^, and 10^8^ EVs/ml). The cells were harvested after 15 min, 4 h, and 24 h of incubation. The Countess™ cell counter and accompanied counting chamber slides (Invitrogen™, Thermo Fisher Scientific) were used to count cells (proliferation) and assess their viability (live, dead, and total cells), using the standard trypan blue technique. To this end, 5 µl of the Trypan Blue Stain (0.4%; Invitrogen™, Thermo Fisher Scientific) was added to 995 µl of the cell solution before counting.

### 2.4 Cell migration assay

A four-well Ibidi silicone culture insert suitable for migration assays was used (Ibidi, Martinsried, Germany). These silicone inserts serve as a migration barrier while seeding the cells and when removed, reveals a defined cell-free gap. The inserts were placed in the middle of each well of a 6-well plate before fibroblasts were seeded at a density of 50000 cells/well and grown for 24 h in 100 μL of standard fibroblast medium. Then, the medium was replaced with starvation medium. After an additional 24 h (48 h after seeding in total), the well inserts were carefully removed, and the medium was replaced with 2 ml of fresh starvation medium with different concentrations of the three EV types (10^6^, 10^7^, and 10^8^ EVs/ml). During the next 24 h, cells were photographed every 4 h (Axiocam 105 color, Carl Zeiss GmbH), and the surface area covered by cells within the defined gap area was measured using NIH ImageJ software (rsb.info.nih.gov/ij).

### 2.5 RT-qPCR

RNeasy Mini kit (Qiagen, Valencia, CA) was used for total RNA extraction from samples collected at 24 h, according to the manufacturer’s instructions. RNA quality and quantity was measured with a NanoDrop spectrophotometer (NanoDrop Technologies Inc., Wilmington, DE). Complementary DNA (cDNA) was synthetized using 200 ng of extracted RNA, the Reverse Transcription Core Kit (Eurogentec, Seraing, Belgium) with a mix of reverse transcriptase enzyme, RNAse inhibitor, MgCl, dNTP, and 10× buffer. Each reaction was performed in a total volume of 30 µl at 25°C for 10 min, then at 48°C for 30 min and terminated by incubation at 95°C for 5 min. Detection of smooth muscle actin type 2 (*ACTA2*), fibroblast activating protein (*FAP*), platelet-derived growth factor receptor (*PDGFR*), Ca^2+^- activated Cl^-^ channel (*ANO1*), and Ca^2+^- activated K^+^ channel (*KCNMA*) was performed using Assay-on-Demand TaqMan Gene Expression probes (AOD, Applied Biosystems, Foster City, CA). Glyceraldehyde 3-phosphate dehydrogenase (*gapdh*) was used as an endogenous control. Each RT-qPCR reaction consisted of 1× AOD mix, 1× qPCR Takyon Low Rox Probe MasterMix dTTP Blue (Eurogentec, Seraing, Belgium) and 10 µl cDNA (diluted 1:2.5 µl with H_2_O) as the template. RT- qPCR reactions were performed in a 96-wells PCR reaction plate on AriaMx Real-Time PCR System (Agilent Technologies, Santa Clara, CA) for 40 cycles (95°C for 15 s and 60°C for 1 min) after an initial 10 min incubation at 95°C. A Non-Template Control (NTC) was included for each run to validate the assay. For each experiment, each sample was run in duplicate. The relative amount of mRNA was standardized to that of *gapdh* mRNA using ΔCq = [Cq (target gene) − Cq (reference gene)] and displayed as 2^(−ΔCq)^.

### 2.6 Statistical analyses

Statistical analysis was performed using SigmaPlot 15.0 (Alfasoft AS, Lillestrøm, Norway). Significance was tested using one way ANOVA. Pairwise multiple comparison procedures were performed with the Tukey test or Holm-Sidak method. Significance was set to *P*<0.05.

## 3 Results

### 3.1 Uptake of cancer-derived EVs by normal oral fibroblasts

**Figure 1:**
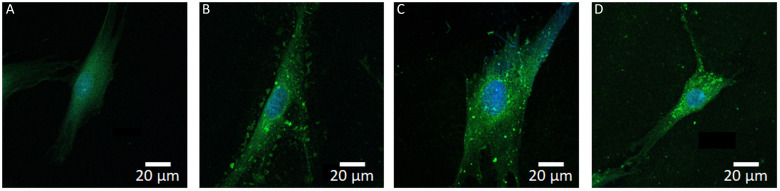
Internalization of fluorescent EVs into oral fibroblasts. The EVs were labeled with green fluorescence (PKH67) and the nuclei were stained blue (DAPI). Samples were imaged with a confocal microscope. From left to right: representative pictures of a control fibroblast **(A)**, and fibroblasts treated with EVs from OSCC **(B)**, PDAC **(C)**, and MBM **(D)** cell lines, respectively.

To investigate the possible uptake of EVs by fibroblasts, the cells were incubated with EVs from the three different cancer-derived cell lines (OSCC, PDAC, and MBM) for 4 h. The EVs were labelled with the green fluorescent PKH67 marker. The distribution of green fluorescence was compatible with cytoplasmic location of the EVs (**Figure 1**). Uptake of EVs was seen in all three cell lines and there were no apparent differences in the uptake between the three cell lines by visual inspection of fluorescence pictures.

### 3.2 Viability and proliferation of normal oral fibroblasts exposed to cancer-derived EVs

**Figure 2:**
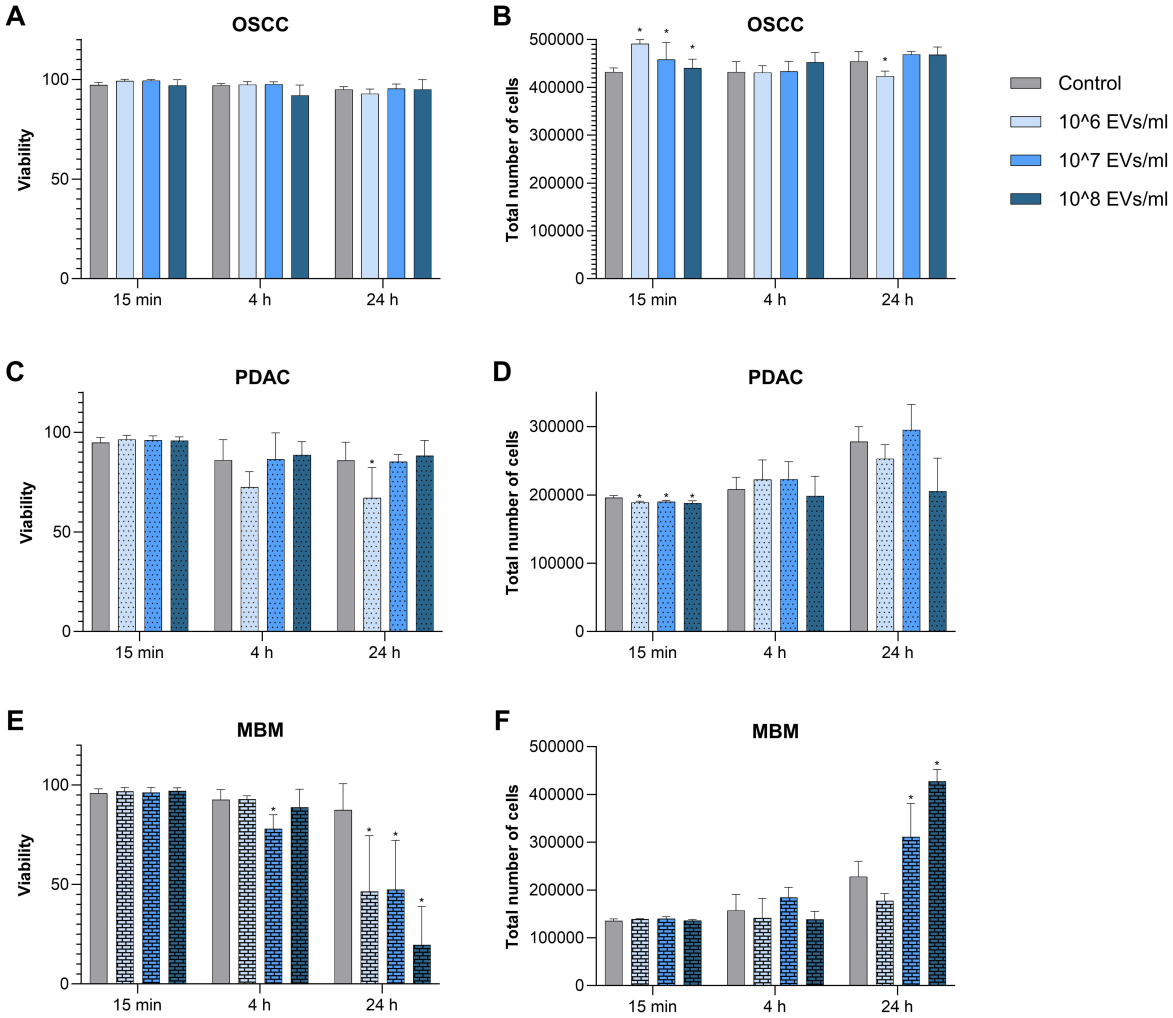
Viability (% living cells) and proliferation (total number of cells) of fibroblasts exposed to EVs from the cancer cell lines OSCC, PDAC, and MBM. The fibroblasts (N=5) were incubated for 15 min, 4 h, and 24 h in starvation medium containing different concentrations of EVs (10^6^, 10^7^, and 10^8^ EVs/ml) from OSCC **(A, B)**, PDAC **(C, D)**, and MBM **(E, F)** cell lines. Counts of living cells and total number of cells were taken from the same samples. (*) *P<0.05*.

To assess whether the fibroblasts were compromised by the uptake of EVs, we measured their viability with and without co-incubation with EVs from OSCC, PDAC, and MBM cell lines for 15 min, 4 h, and 24 h (**Figure 2**). Viability was measured as % living cells. The addition of the OSCC EVs did not affect fibroblast viability. However, EVs of PDAC origin caused a significant decrease in fibroblast cell viability at 10^6^ EVs/ml for 24 h (22%). For EVs from the MBM cell line, a significant decrease in viability was noted for 10^7^ EVs/ml at 4 h (16%), and for all EV concentrations at 24 h (47%, 46%, and 78% at 10^6^, 10^7^, and 10^8^ EVs/ml, respectively).

To assess the effect of EVs on fibroblast proliferation, the change in the total number of cells was measured at different EV concentrations and incubation times. OSCC EVs significantly increased cell proliferation at 15 min for all three concentrations (14%, 6%, and 2% respectively) and decreased cell proliferation at 24 h (10^6^/ml) (6.8%) (**Figure 2**). This is in contrast to the PDAC EVs, where all concentrations significantly decreased cell proliferation at 15 min (3%, 3%, and 4%), while there were no differences at 4 h and 24 h. For MBM, EVs at the two highest doses (10^7^/ml and 10^8^/ml) significantly increased proliferation of the fibroblasts at 24 h (36% and 87%, respectively), while there were no significant changes at the other time points.

### 3.3 EVs from PDAC and MBM cells increase migration of normal oral fibroblasts

**Figure 3:**
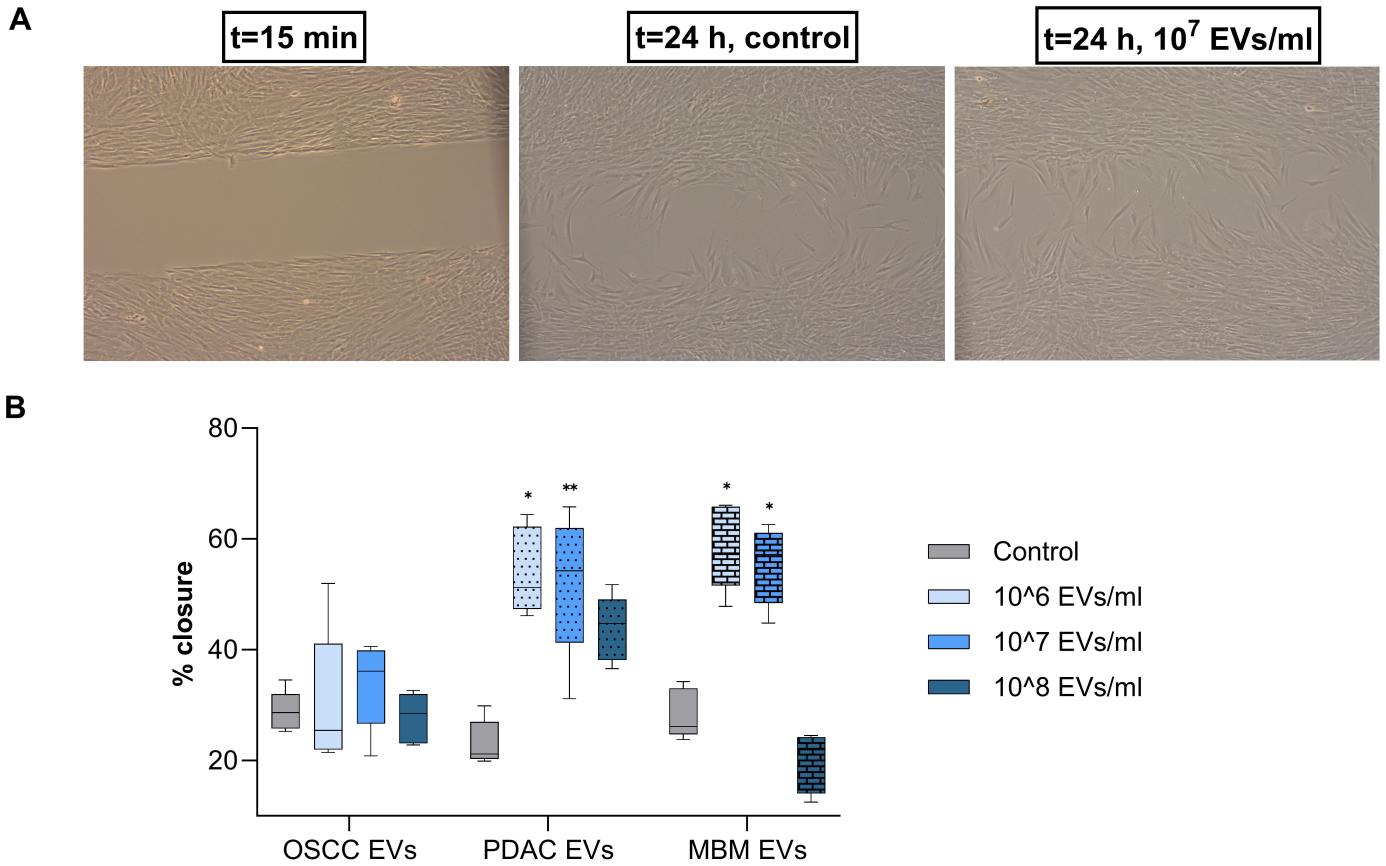
Migration of fibroblasts exposed to EVs from OSCC, PDAC, and MBM cell lines. **(A)** Representative light microscope pictures of fibroblasts at 15 min and after 24 h of incubation with/without (control) OSCC-EVs. **(B)** Box plots presenting the average wound closure (%) for fibroblasts (N=5) exposed for 24 h to EVs of OSCC, PDAC, and MBM origin in different concentrations: 10^6^, 10^7^, and 10^8^ EVs/ml. (*) *P*<0.05; (**) *P*<0.01.

A classical wound-healing assay was performed to assess whether cancer-derived EVs induced CAF-like migratory behavior. Fibroblasts gradually migrated into the central cell free area during the 24 h observation period (**Figure 3A**). Increased fibroblast migration, observed as an improved wound closure percentage, was induced by PDAC and MBM EVs, while the differences observed for the OSCC EVs were not statistically significant (**Figure 3B**). For the PDAC EVs, the 10^6^/ml and 10^7^/ml concentrations induced a significantly increased wound closure (134% and 125%, respectively). Also, the EVs of MBM origin showed a significant stimulation of wound closure at 10^6^ and 10^7^ EVs/ml (106% and 95%, respectively).

### 3.4 EVs from OSCC, PDAC, and MBM cell-lines affect mRNA expression of CAF biomarkers in normal oral fibroblasts

**Figure 4:**
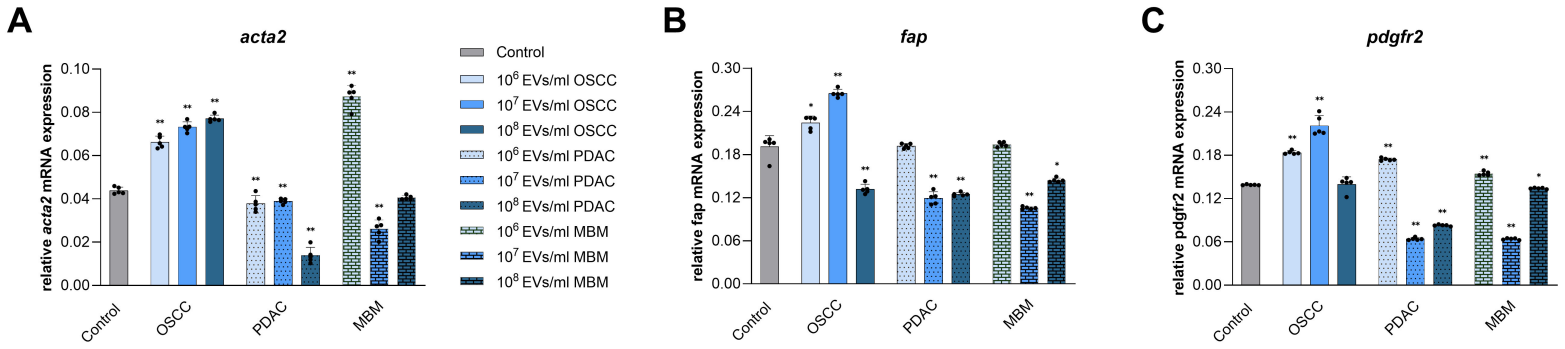
mRNA expression of CAF biomarkers in fibroblasts exposed for 24 h to EVs from OSCC, PDAC, and MBM cell lines. Bar charts representing average *acta2* **(A)**, *fap* **(B)**, and *pdgfr* **(C)** mRNA expression levels in fibroblasts (N=5) exposed to cancer EVs (106, 107, 108 EVs/ml), relative to the reference gene *gapdh* (2ΔCq). (*) P<0.05; (**) P<0.01.

To assess potential CAF transformation of normal fibroblasts, we used ACTA2, FAP, and PDGFR, three well established biomarkers for CAFs. EVs from all three cell lines affected mRNA expression of the CAF biomarkers in the fibroblasts at 24 h exposure, and the scale of the impact depended on the concentration of vesicles added (**Figure 4**). EVs from the OSCC cell line significantly increased expression of *acta2* at all concentrations (51%, 67%, and 76%), while EVs from the PDAC cell line decreased this expression at all concentrations (14%, 11%, and 68%) (**Figure 4A**). EVs from MBM cells increased *acta2* expression at 10^6^/ml (99%) and reduced it at 10^7^/ml (40%). *Fap* expression was increased by OSCC EVs at 10^6^/ml and 10^7^/ml (17% and 39%), while the 10^8^/ml concentration had a decreasing effect (31%). PDAC EVs reduced *fap* expression at both 10^7^/ml and 10^8^/ml (37% and 35%). Similarly, MBM EVs significantly reduced *fap* expression at 10^7^/ml and 10^8^/ml EVs (45% and 24%, respectively) (**Figure 4B**). OSCC EVs upregulated *pdgfr* expression at 10^6^/ml and 10^7^/ml concentrations (32% and 59%, respectively), while both PDAC and MBM EVs increased *pdgfr* expression at 10^6^/ml (25% and 11%, respectively) but decreased it at 10^7^/ml (54% and 54%, respectively) and 10^8^/ml (40% and 3%, respectively) (**Figure 4C**).

### 3.5 EVs from OSCC, PDAC, and MBM cells affect mRNA expression of Ca^2+^-activated ion channels in normal oral fibroblasts

**Figure 5:**
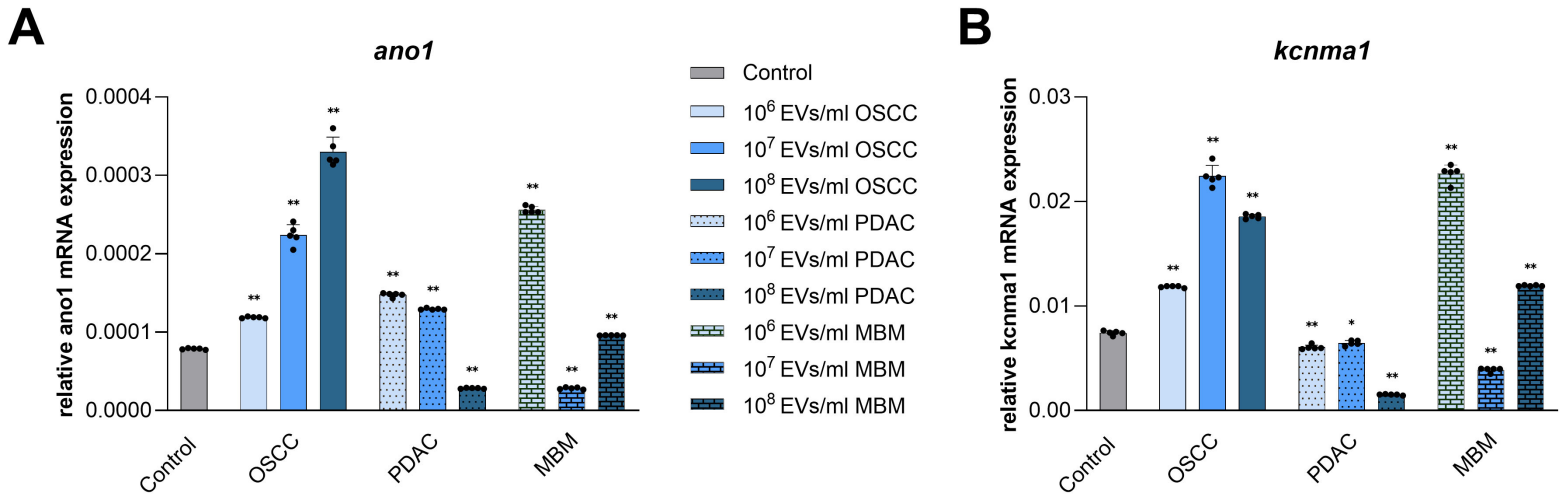
mRNA expression of Ca2+-activated ion channels in fibroblasts exposed for 24 h to EVs from OSCC, PDAC, and MBM cell lines. Bar charts representing average a*no1* **(A)** and *kcnma* **(B)** mRNA expression levels in fibroblasts (N=5) exposed to EVs (106, 107, 108 EVs/ml), relative to the reference gene *gapdh* (2ΔCq). (*) P<0.05; (**) P<0.01.

Among several ion channels implicated in cancer development and CAF transformation, the Ca^2+^-activated Cl^-^ channel ANO1 and Ca^2+^-activated K^+^ channel KCNMA (BK) were chosen to investigate their expression in normal oral fibroblasts under the influence of cancer-derived EVs. Dependent on the EV concentration and their origin, both stimulation and inhibition of mRNA expression of the selected Ca^2+^-activated ion channels were observed in fibroblasts (**Figure 5**). EVs from the OSCC cell line significantly increased expression of *ano1* transcripts in fibroblasts at all concentrations (50%, 184%, and 319%). EVs from PDAC induced an increase at 10^6^/ml and 10^7^/ml (88% and 65%, respectively), while 10^8^/ml inhibited *ano1* expression (64%). EVs from the MBM cell line significantly increased *ano1* at 10^6^/ml and 10^8^/ml (225% and 22%, respectively), but decreased the expression at 10^7^/ml (64%) (**Figure 5A**). Regarding *kcnma*, EVs from the OSCC cell line increased expression at all concentrations (59%, 202%, and 150%). On the contrary, PDAC EVs decreased the expression of *kcnma* at all concentrations (19%, 13%, and 80%) (**Figure 5B**). EVs from the MBM cell line significantly increased *kcnma* expression in fibroblasts at 10^6^/ml and 10^8^/ml (205% and 60%, respectively), while decreasing it at 10^7^/ml (48%).

## 4 Discussion

The current study was performed to reveal effects of cancer-derived EVs of different origin on the phenotype of normal oral fibroblasts (NOFs). NOFs were exposed to EVs derived from an oral squamous cell carcinoma (OSCC) cell line, representing “local” oral cancer. In addition, NOFs were exposed to EVs derived from cell lines of two “distant” cancers, a pancreatic adenocarcinoma (PDAC) and a melanoma brain metastasis (MBM). Although all three cancer-derived EVs seemed to be taken up by the NOFs, the following NOF response was dependent of the type of cancer EV in addition to the EV concentration and duration of EV exposure. This indicates that fibroblasts in the oral cavity also can receive and process signals from cancer EVs derived from cancers at other sites than the oral cavity. This finding could be of importance for further studies of the role of EVs in e.g., metastatic processes and their role in how host cells at the metastatic site can be orchestrated by the arriving cancer cell EVs.

In NOFs, cytosolic labelling consistent with internalization of EVs from OSCC, PDAC, and MBM was observed after EV exposure. Therefore, it is justified to assume that the observed changes in NOFs were due to EV uptake and internalization of their molecular cargo. There were no apparent differences between the origin of the EVs and the amount of EVs taken up by NOFs.

Although EV uptake in NOFs was not influenced by EV origin, EV origin affected NOF viability and proliferation. With regard to proliferation, OSCC EVs immediately increased NOF proliferation, but after 24 h of exposure a decreased proliferation was detected. The response to PDAC EVs was opposite, with an immediate decreased proliferation at 15 min. Increased proliferation was detected after 24 h of exposure of the MBM EVs, while there was no effect at the earlier time points. A report on EVs derived from head and neck squamous cell carcinoma (HNSCC) cells showed enhanced oropharyngeal fibroblast proliferation (25). Also, EVs in gastric juice from gastric cancer patients were found to promote proliferation of normal fibroblasts (26). This is in agreement with our observation of the response in NOFs when exposed to cancer EVs. Although several studies report on increased fibroblast proliferation after exposure to cancer EVs, our current results indicate that this might not always be the case. We found that the influence of cancer EVs on fibroblast proliferation depended on cancer origin, as EVs derived from site-specific and distant cancers affected NOFs differently, which is an important point to consider in future studies.

In terms of viability of NOFs exposed to EVs, again the origin of the vesicles determined the response. No effect on viability was observed for EVs derived from the site specific cancer cells (OSCC), while EVs from the non-site specific cancers PDAC and MBM contributed to decreased viability of NOFs. Our results indicate that the level of decrease in viability was also dependent on the EV dose and duration of exposure. An increasing dose and increasing exposure time of EVs from non-site specific cancers might have a negative impact on NOFs viability. Whether the reduced viability at high doses of EVs are a particular effect of the EV cargo, or simply a result of vesicle overload, needs to be further assessed. Nevertheless, reports on EV effect on cell viability are diverse. Exemplary, EVs from metastatic isogenic colorectal cancer had a cytotoxic influence on some populations of macrophages (27). While, treatment of an epithelial kidney cell line with HeLa vesicles resulted in a significant increase in cell viability (28).

Generally, increased migration of NOFs was induced by EVs from PDAC and MBM cells. The effect on migration was dose-dependent, and it was observed that the 10^6^/ml and 10^7^/ml concentrations were more effective than the 10^8^/ml concentration. Furthermore, MBM EVs decreased migration capacity at 10^8^/ml, which suggest that the highest EV concentration may be a supra-physiological dose (29, 30). The obtained data are consistent with a report where HNSCC EVs stimulated migration of oropharyngeal normal fibroblasts (25). Increased migration induced by EVs has also been demonstrated in cancer cells stimulated by EVs from the same cancer type such as OSCC cell-derived EVs, promoting OSCC cell migration (31). Additionally, metastatic skin melanoma EVs accelerated the migration of primary melanoma cells but also increased the growth and migration of normal keratinocytes and metastatic cells (32, 33). Migration of immortalized human bronchial epithelial cells under the influence of human non-small cell lung carcinoma derived EVs was also enhanced (34). Interestingly, in our study only EVs from cancers originating in another site than the oral mucosa stimulated NOF migration (i.e., no effect of OSCC EVs). Part of the explanation could perhaps be that there is a need for metastasizing cancer cells to attract local fibroblasts and develop a tumor stroma at their new tumor site. Although the molecular background for the changes in cell function such as proliferation and migration may relate to the EV cargo, the mechanisms behind are still under investigation. It is, however, notable that EV-associated long non-coding RNAs may bind to particular proteins of recipient cells upon being internalized via EV uptake (28), and could, thereby, be one of the mechanisms behind the EV-induced changes of cell function.

To verify if the changes detected in proliferation, viability, and migration of the NOFs exposed to cancer EVs may constitute a possible phenotype switch towards CAFs, expression of the well-established CAF biomarkers ACTA2, FAP, and PDGFR was determined at the mRNA level. All of the CAF biomarkers were significantly upregulated in NOFs independent of EV origin, although a differential expression level of the CAF biomarker mRNAs were seen depending on the origin of the cancer EVs. Specifically, in the current study the PE/CA-PJ49/E10 cell-line was used to produce OSCC EVs, and the *fap* results overlap with an increase in *fap* expression reported in NOFs treated with EVs derived from another OSCC cell line (CAL27) (35). Among OSCC CAFs, one subpopulation has been identified as myofibroblast-associated CAFs due to the high expression of ACTA2, TAGLN, MMP11, MYL9, POSTN, TPM1, and TPM2 while another subpopulation termed inflammatory CAFs was characterized with highly expressed PDGFRA and lack of ACTA2 (36). Single-cell studies of PDAC CAFs show that there are multiple subpopulations of CAFs that can be characterized with specific sets of expressed genes, and the composition of those subpopulations may vary due to stage of tumorigenesis (37, 38). During late PDAC progression, CAF subsets expressing myofibroblast markers such as *acta2* and *tagln49* are dominant (39), however in other subsets, *acta2* up-regulation is not reported (40, 41). In PDAC, CAFs are divided into FAP^+^ or FAP^-^ and it is the presence of FAP expression itself that is considered as a CAF biomarker rather than the expression level. Moreover, it is reported that FAP^+^ CAFs are also positive for PDGFR (42). In CAFs originating from MBM, expression of the biomarkers α-SMA, PDGFR2, and FAP is much higher than in normal fibroblasts (43). Taken together with the results of proliferation, viability, and migration, the mRNA results support the hypothesis that NOFs exposed to EVs of different cancer origin can change their phenotype, and that the nature of this effect depends on the type of cancer.

The potential biomarkers ANO1 and KCNMA1, that are Ca^2+^-activated ion channels dysregulated in several cancers, were also studied since they play an important role in regulation of cell cycle, proliferation, and migration (19). Interestingly, changes in *ano1* and *kcnma1* expression were similar to the changes in the established CAF biomarkers we analyzed. The direction of change and dose-dependency of the response for the Ca^2+^-activated ion channels and CAFs biomarkers were consistent for each source of EVs. In the current experiment, NOF exposure to OSCC EVs resulted in a dose-dependent increase in *ano1* expression, while PDAC and MBM EVs stimulated *ano1* expression in two out of three concentrations. In OSCC, ANO1 expression promotes cancer cell migration and correlates with occurrence rate, progression, and metastasis (44). ANO1 is also considered a diagnostic biomarker and potential treatment target in OSCC (44, 45). Studies performed on several PDAC cell lines show that ANO1 is over-expressed at both mRNA and protein levels compared to a normal pancreatic ductal epithelium cell line (46). Little is known about the occurrence and putative role of ANO1 in fibroblasts.

Our experiments further revealed that OSCC EVs similarly caused an increase in *kcnma1* expression in all three concentrations. MBM EV effect on the *kcnma1* expression was dose-dependent and both 10^6^/ml and 10^8^/ml EV concentrations caused increase, similar to the results for *ano1*. So far, KCNMA1, has mostly been investigated in fibroblasts concerning its role in fibrotic diseases, and those studies report up-regulation of *kcnma1* in pathological conditions that contribute to cell activation, migration, and proliferation through the regulation of cell membrane potential (47, 48). KCNMA1 is also upregulated in several cancer types, such as prostate, breast, ovarian, and endometrial cancer, where it is involved in cancerous behavior like cell proliferation and migration (49, 50). These findings all fit well with our obtained results on proliferation and viability as well as the results on the genes encoding Ca^2+^ ion channels. To our knowledge, there is no evidence in the literature of differential expression of these two channels between different subpopulations of CAFs. However, based on our observation that they seem to be upregulated in fibroblasts exposed to cancer-derived EVs, they may be eligible as CAF biomarkers in the future. It would be also interesting to investigate whether their expression is more pronounced in particular subsets of CAFs.

In summary, the results revealed that NOFs are able to take up EVs from three different cancer types, one of origin in the same tissue as where the fibroblasts are found (local) and two cancers developed at other sites (distant) than the oral mucosa. The NOF response was dependent of the type of cancer-derived EVs, EV concentration, and duration of EV exposure. However, in most cases the influence of EVs on fibroblast proliferation, viability, migration, and expression of CAF biomarkers was pointed in the same direction for EVs derived from each specific cancer type. This finding highlights the significance of EV origin on NOF response. It also supports the statement that the effect of EV uptake should be evaluated for each specific cell type of interest and origin of EV-derived cells. Nevertheless, our results indicate how exposure to cancer EVs of different origin can drive NOFs towards a pro-tumorigenic phenotype particularly of interest for metastatic disease.

## Data Availability

The raw data supporting the conclusions of this article will be made available by the authors, without undue reservation.
