# Supplementary material for: Extracellular vesicles from cancer cell lines of different origins drive the phenotype of normal oral fibroblasts in a CAF-like direction
Source: Front Oncol. 2024 Sep 24;14:1456346. doi: 10.3389/fonc.2024.1456346 (PMC11458688; doi:10.3389/fonc.2024.1456346)
Supplement: Supplementary file 1 [file DataSheet1.pdf]

Supplementary Material

Supplementary Table 1. Summary of results.

**EV source**	**OSCC**			**PDAC**			**MBM**		
**EV concentration [EVs/ml]**	**10^6**	**10^7**	**10^8**	**10^6**	**10^7**	**10^8**	**10^6**	**10^7**	**10^8**
**Viability 15 min**	+	+	+/-	+	+	+	+/-	+/-	+/-
**Viability 4 h**	+/-	+/-	-	-	+/-	+	+/-	**-***	-
**Viability 24 h**	-	+/-	+/-	**-***	+/-	+	**-***	**-***	**-***
**Proliferation 15 min**	**+***	**+***	**+***	**-***	**-***	**-***	+/-	+/-	+/-
**Proliferation 4 h**	+/-	+/-	+	+	+	-	-	+	-
**Proliferation 24 h**	**-***	+	+	-	+	-	-	**+***	**+***
**Migration 24 h**	+	+	+/-	**+***	**+****	+	**+***	**+***	-
acta2	**+****	**+****	**+****	**-****	**-****	**-****	**+****	**-****	-
fap	**+***	**+****	**-****	+/-	**-****	**-****	+/-	**-****	**-****
pdgfr	**+****	**+****	+/-	**+****	**-****	**-****	**+****	**-****	**-***
ano1	**+****	**+****	**+****	**+****	**+****	**-****	**+****	**-****	**+****
kcnma	**+****	**+****	**+****	**-****	**-***	**-****	**+****	**-****	**+****

Abbreviations: + indicates increase in analyzed parameter, while - indicates decrease; +/- means that there was no change between control and EV exposed group. Statistically significant changes are marked in bold with * ($p \leq 0.05$) or ** ($p \leq 0.01$).
